# *Tenebrio molitor* Could Be an Efficient Pre-Treatment Bioagent for Polystyrene Initial Deterioration and Further Application of *Pleurotus eryngii* and *Trametes versicolor* in Microplastic Biodegradation

**DOI:** 10.3390/polym17131772

**Published:** 2025-06-26

**Authors:** Slobodan Stefanović, Milena Dimitrijević, Dragosav Mutavdžić, Kristina Atlagić, Slobodan Krnjajić, Žaklina Marjanović

**Affiliations:** 1 Faculty of Applied Ecology FUTURA, Metropolitan University, Požeška 83a, 11000 Belgrade, Serbia; slobodan.stefanovic@futura.edu.rs; 2 University of Belgrade—Institute for Multidisciplinary Research, Kneza Višeslava 1, 11000 Belgrade, Serbia; milena.dimitrijevic@imsi.bg.ac.rs (M.D.); gane@imsi.bg.ac.rs (D.M.); slobodan.krnjajic@imsi.bg.ac.rs (S.K.); 3 Department of Physiology and Biophysics, Faculty of Biology, University of Belgrade, Studentski Trg 16, 11158 Belgrade, Serbia; kristina.tesanovic@bio.bg.ac.rs

**Keywords:** polystyrene biodegradation, microplastic biodegradation, *Tenebrio molitor*, *Pleurotus eryngii*, Trametes versicolor

## Abstract

Plastic is a major organic pollutant globally but has only recently been recognized for its recalcitrant nature and resistance to degradation. Although vast amounts of plastic debris are overwhelming the planet, the search for solutions to its degradation has only recently begun. One of the most well-known agents of plastic biodegradation is the larvae of *Tenebrio molitor*, which can alter the structure of polymers like polystyrene. However, while this insect can cause deterioration, its frass, which still consists of polystyrene microplastics, remains a problem. We investigated whether this frass could be further degraded by strains of white rot fungi, specifically *Pleurotus eryngii* and *Trametes versicolor*. We introduced two PS derivatives (styrofoam and stirodure) to the fungi in liquid media and evaluated oxidative metabolism enzymes (laccase, Mn-peroxidase, lignin-peroxidase) activities, and the phenolic products of the potential aromatic polymer degradation in the media. Finally, we evaluated FTIR spectra to determine if we could detect changes in polystyrene molecule degradation. Both fungi produced high amounts of enzymes, particularly when the polystyrene was present. Large quantities of phenolic substances were simultaneously detected, some associated with polystyrene degradation. FTIR spectra of different polystyrene products confirmed species-specific mechanisms for their degradation by experimental fungal strains.

## 1. Introduction

Plastic polymers are a major problem worldwide because they accumulate as hardly degradable solid waste on the Earth’s surface [1]. There are various forms of plastics, of which polystyrene (PS) has been one of the most produced and used in the last decade. In 2016 alone, the global production of PS was around 14.7 million metric tons per year [2], while in Europe, it represents 6–7% of total annual plastic production [3]. PS production has consistently increased due to its excellent properties for use as a building material, packaging foam, food packaging, and disposable cups, plates, and cutlery. However, most of these products, usually after a single use, sooner or later end up in landfills and nature. In its basis, PS is a large and stable polymer consisting of aromatic styrene monomers with a large molecular weight and high hydrophobicity [4,5].

PS products are easily physically disintegrated into small particles, which is why they are the most prominent producers of microplastics (MPs) in the environment. MPs usually end up in the soil around construction fields, garbage disposals, or anywhere people dump them. Like other plastics, due to the very stable C-C bonds, the degradation of PS is generally a very slow process lasting over long periods [4].

However, since the PS is an organic polymer similar to lignin, enzymatic systems of organisms like bacteria and fungi, as well as some insect larvae (e.g., *Tenebrio molitor* L.), are able to perform its biodegradation [1,6]. Biodegradation is a complex process that transforms plastic objects and particles, as well as large polymers, into smaller units mediated by biological factors [7,8]. This process is assumed to be divided into four stages: biofilm formation, biodeterioration, biofragmentation, and assimilation [9]. The first step in degrading particles made of high molecular weight and long-chain polymers is the weakening of their structure. Several factors can influence plastic biodegradation, including environmental conditions, exposure to UV light, the hydrophobicity of the exposed area, the chemical structure, crystallinity grade, and elasticity [10,11,12]. Organisms like microorganisms or insect larvae may initiate the biodegradation of some plastics, which, at their final stage, become a food source [6]. Thus, biodegradation is an environmentally friendly and cost-effective method of degrading plastics, in contrast to photodegradation and thermal oxidation, which are classified as environmentally harmful methods.

White rot fungi are known as the most efficient degraders of organic matter in nature, producing different enzymes that are used in diverse processes leading towards efficient and complete degradation of recalcitrant natural polymers like lignin [13,14,15,16]. Lignin is comprised of three types of phenyl–propane (aromatic) units bonded to one another in different ways [17]. In the presence of a potential substrate, white rot fungi excrete exoenzymes that attack the surface of the material and gradually degrade adequate polymers [18]. The most well-known enzymes for the biodegradation of lignin are laccases (Lac) and peroxidases, presumably Mn- (MnP), lignin- (LiP), and versatile peroxidases [19,20,21], which have different substrate ranges depending on their respective redox potentials [22,23]. All three enzymes are non-specific towards substrates with high redox potentials—they attack the phenolic, but also non-phenolic subunits of lignin [21,24,25,26]. Laccases produced by white rot basidiomycetes have high redox potential and diverse catalytic properties, so they can perform diverse biotechnologically challenging processes [27,28,29]. Laccases use oxygen as an electron acceptor to oxidize phenolic and non-phenolic compounds [30,31,32]. MnP catalyses oxidation of Mn^2+^ to Mn^3+^ in a H_2_O_2_-dependent reaction, attacking both phenolic and non-phenolic compounds [33], while LiP catalyses H_2_O_2_-dependent oxidative depolymerization of lignin [34].

Since the first step in plastic biodegradation is the formation of biofilms on the material surface, filamentous fungi that have fast hyphal apical and multi-directional growth [35] appear to be very suitable for performing such a process. Additionally, they are capable of synthesizing small globular proteins (<20 kDa) known as hydrophobins that form amphipathic monolayers at interfaces with the substrates, thus forming compact fungus–enzyme–substrate relationships [36].

Similarly to lignin degradation, in plastic biodegradation processes, the fungal mycelia overgrow the plastic and produce enzymes that break down the high molecular weight polymers into low molecular weight units, or even mineralize them totally [1]. Under the scanning electron microscope (SEM), the damage caused by fungi on the plastic surfaces can be easily detected [37,38]. White rot fungi such as *Pleurotus eryngii* (DC.) Quél. and *Trametes versicolor* (L.) Lloyd are known to be efficient not only in the biodegradation of lignin but also in the biodegradation of plastic polymers [1]. *P. eryngii* was able to degrade di-2-ethylhexyl phthalate (DEHP) plastics due to the production of MnP [39]. Similarly, *T. versicolor* degraded high molecular weight polyethylene with the enzyme MnP [40] and the lignin–polystyrene copolymer by producing oxidative enzymes [41]. Even though high resistance of the C–C backbones in PS polymer makes its degradation difficult for fungi, Milstein et al. [41] recorded SEM-confirmed polymer overgrowth, as well as biodegradation of the lignin–polystyrene copolymer by basidiomycete white rot fungi *Phanerochaete chrysosporium*, *Trametes versicolor*, and *Pleurotus ostreatus*, accompanied by the production of LiP and Lac.

However, for initiating the PS degradation process, fungi are likely not efficient because their exoenzymes are too large to penetrate deeply into the material, so they can only act on the polymer’s surfaces, eventually causing surface erosion [42]. For this reason, few studies have used pre-treatments like UV light, ozonation, chemical oxidants, and thermal exposures to oxidize the polymer and create reactive functional groups (carbonyl/carboxyl/hydroxyl groups) to enable fungal enzymes to act [43,44]. Oxidative enzymes secreted by fungi gradually corrode the hydrophobic inert surface of PS, making it rough and more accessible for further colonization by fungal mycelia [45]. Additionally, in our previous work, we concluded that the efficiency of lignolytic enzymes depends on the substrate’s surface—the larger the surface area of the substrate (smaller particles), the more exposed it will be to the exoenzymes, and the more efficient the degradation will be [16].

In addition to the fungal enzymes’ ability to degrade plastics, the insect larvae of *T. molitor* are also capable of degrading PS in their digestive tract due to their gut microflora [6,46,47]. Styrene, the building block of PS, could be metabolized and utilized by bacteria [48], including insect gut bacteria. Insects can survive long periods (even months) solely feeding on plastics and even transform from larvae to imago stadium in such conditions (S. Krnjajić, unpublished). The darkling beetle *Plesiophthalmus davidis* larvae could survive on PS as a feed and degrade PS in their gut via depolymerization and oxidation [49]. However, after the polystyrene consumption, insects reveal frass that consists of small particles of undegraded plastic material. These particles are app. 0.2 mm in size, therefore belonging to the MP (Krnjajić et al., unpublished), implying that insects could be good bioagents for the deterioration of PS but are incapable of complete biodegradation.

The plastic biodegradation process is very slow and not easy to observe under the experimental conditions. Several studies have assessed the plastic polymer degradation abilities of various microorganisms using Fourier transform infrared spectroscopy (FTIR) [37,50,51,52] by comparing the spectra obtained from the plastics before and after treatment with fungi. The graphical representations of the initial spectrum and the difference spectrum show the regions/absorbances that were subjected to chemical changes, including the formation or disappearance of functional groups on the surface of a polymer upon microbial degradation [53]. The appearance of new peaks, along with the disappearance and shifting of peaks attributed to the stretching and bending vibrations of different functional groups, is assumed to confirm the structural changes that occurred on the polymer surface upon biodegradation [37,50,51,52]. Additionally, it is assumed that the oxidoreductases (laccases and peroxidases) are involved in PS degradation into smaller molecules, such as oligomers, dimers, and monomers [54,55]. Similarly to lignin, these products can be detected using high-pressure liquid chromatography (HPLC) for detecting aromatic products of fungi-mediated degradation of PS [16]. This technique can be combined with FTIR spectra to demonstrate the biodegradation of PS by biological agents.

In the present study, we grew the insect larvae of *T. molitor* on two types of PS: styrofoam, expanded polystyrene (EPS), and styrodure, extruded polystyrene (XPS). The processed PS in the form of obtained larval frass, which was the size of the MP and supposedly having undergone a certain level of polymer destruction in the insect gut, was used as a substrate for the cultivation of two species of white rot fungi, *Pleurotus eryngii* and *Trametes versicolor*, in the liquid medium. The activities of laccase, lignin, and manganese peroxidases in the growth media were measured to determine their possible involvement in PS microplastic degradation. To assess the influence of *Tenebrio molitor* on PS degradation, as well as the influence of two fungal species on PS microplastic particles, we used Fourier transform infrared spectroscopy (FTIR) to determine whether there were structural changes in the larvae- and fungi-treated EPS and XPS. Finally, UHPLC/Orbitrap MS analysis was performed to identify phenolic compounds as potential products of plastic degradation in fungal media.

We hypothesized that *Tenebrio molitor* consumption of intact EPS and XPS could be an efficient pre-treatment for the formation of MP particles and the initial stage of the change in PS structure (deterioration), which will enable an efficient approach of fungal enzymes to the polymer and cause its further biodegradation. Such an experimental setup could lead to an improved and innovative bio-based process for PS biodegradation.

## 2. Materials and Methods

### 2.1. Insect Treatment

**Figure 1:**
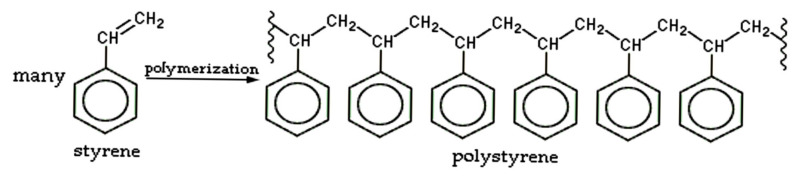
The structure of polystyrene (taken from Yanto et al.) [56].

**Figure 2:**
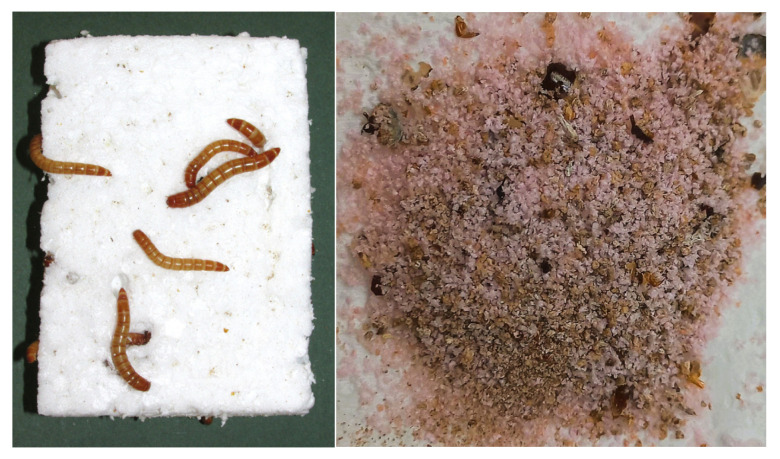
Larvae of *Tenebrio molitor* feeding on EPS (**left**) and frass of *T. molitor* that was fed on XPS before H_2_O_2_ treatment (**right**).

The specimens of the insect species *Tenebrio molitor* L. have been grown and maintained in the laboratory of the University of Belgrade Institute for Multidisciplinary Research on the natural food source (wheat flour), and the obtained larvae were used in the experiment. Twenty larvae L5 stage were placed in each of the two plastic boxes with a volume of 5 L, and two types of polystyrene foam were added separately in each box as the sole food source: styrofoam, expanded polystyrene (EPS), and styrodure, extruded polystyrene (XPS) (Figure 1). Both types of polystyrene were in the form of a thick sheet cut into irregular cube-shaped pieces a few centimeters in size (Figure 2). Larvae in two plastic boxes were incubated in the dark at room temperature, around 23–25 °C and 70% humidity for 6 weeks. For the time given, 80% of larvae survived and were transformed into imago, while 20% did not manage to complete the entire life cycle and eventually died. The insect larvae’ frass was collected, analyzed, and used as a substrate in experiments with fungi (Figure 2).

### 2.2. Fungal Treatment

*Trametes versicolor* (L.) Lloyd strain SerBC was isolated from the fruiting body originating from the forest in northern Serbia, while *Pleurotus eryngii* (DC.) Quél. strain EspCF was received from the mycelia-producing company Cultivos Forestales, Torre de Las Arcas, Spain. The mycelial cultures of *P. eryngii* and *T. versicolor* were maintained in Petri dishes on malt extract agar in the dark at room temperature. To confirm the strains’ taxonomic determination, DNA was isolated from mycelia and used for PCR-based amplification with standard primers for fungi (ITS1F and ITS4) and applying standard protocols [57]. The PCR products were sent for Sanger sequencing to Microsyth, Vienna, and the obtained sequences were compared with existing ones in the NCBI database using the BLASTn option from the site [58].

For the experiment on MP degradation by both fungal species, the liquid medium containing 15 g/L of malt extract and 5 g/L of peptone was used. In 100 mL Erlenmeyer flasks, 50 mL of liquid medium was added and sterilized in an autoclave at 114 °C for 30 min. Liquid media were inoculated with 5 one-week-old mycelial plugs of both fungi species separately per flask. Insect larval frass was treated with 30% hydrogen peroxide to decompose organic matter and bacteria from the larvae’s intestinal tract, carefully washed with sterile water, dried, and added to the liquid cultures of both fungal species, 300 mg per Erlenmeyer flask. Frass of insect larvae, grown on two types of polystyrene, was added separately. In addition to the experimental treatments, appropriate controls were prepared: liquid medium with added insect larval frass that was not inoculated with fungal mycelia and liquid fungal cultures of fungi to which no plastic substrate was added. All the experimental treatments were incubated at 30 °C in the dark for 30 days.

### 2.3. Sample Preparation

Control of EPS and XPS, as well as experimental insect frass treated with 30% hydrogen peroxide, were analyzed directly using Fourier transform infrared spectroscopy (FTIR).

The fungi-treated sample preparation for analysis involved separating the liquid and solid phases on filter paper using a vacuum pump. Solid phases, containing fungal mycelia and polystyrene particles, were lyophilized and stored in a freezer at −20 °C until FTIR analysis. Liquid phases in the amount of 4185 g were centrifuged for 30 min. A total of 3 mL of supernatant was collected and used for the analysis of the ligninolytic enzyme activity. The rest was lyophilized, stored in a freezer at −20 °C, and used for high-performance liquid chromatography (HPLC) analysis.

### 2.4. Laccase Activity

The determination of laccase activity was carried out according to the protocol of [59], which is based on the oxidation of guaiacol. The reaction mixture of 10 mM sodium acetate buffer, pH 5, 2 mM guaiacol, and the enzyme source was made. After incubation of the reaction mixture at 30 °C for 15 min, the absorbance of oxidized guaiacol was measured at 470 nm (2501 PC Shimadzu, Kyoto, Japan). For the calculation of enzyme activity (U/mL), the extinction coefficient of guaiacol was taken to be ε = 6.74 L/(mmol × cm) as reported by Hosoya [60].

### 2.5. Mn Peroxidase Activity

Determination of Mn peroxidase activity was performed according to the previously published protocol of Cascielo et al. [61], which is based on ABTS oxidation. The reaction mixture of 0.5 mM ABTS and 0.16 mM MnCl_2_ in a 40 mM sodium citrate buffer, pH 4.5, was prepared, and the reaction was initiated with the addition of 0.05 mM H_2_O_2_. The absorbance was measured at 420 nm (2501 PC Shimadzu, Kyoto, Japan) against distilled water as the reference mixture (ε = 36,000 M^−1^cm^−1^). For the enzyme activity calculation, it was assumed that one unit of enzyme activity (U) corresponds to the amount of enzyme needed to oxidize 1 μmol of ABTS per minute at 25 °C.

### 2.6. Lignin Peroxidase Activity

The determination of lignin peroxidase activity was performed according to the previously published protocol [62], which is based on the oxidation of Azure B dye. The reaction mixture, consisting of a 125 mM sodium tartrate buffer (pH 3.0), 0.16 mM azure B, and enzyme filtrate in a total volume of 1 mL, was initiated with the addition of 2 mM H_2_O_2_. The decrease in absorbance was measured at 651 nm (ε = 48.8 L/mmol·cm; 2501 PC Shimadzu, Kyoto, Japan). One unit of enzyme activity (U) was expressed as a ΔA of 0.1 unit per minute per ml of the culture filtrate.

### 2.7. UHPLC/Orbitrap MS Analysis

The phenolic compounds were identified in methanol extracts using an LC/MS (Thermo Scientific™ Vanquish™ Core HPLC system, Waltham, MA, USA) coupled to the Orbitrap Exploris 120 mass spectrometer (San Jose, CA, USA). All LC/MS parameters and procedures for data acquisition, processing, and analysis are explained in detail in Stojkovic et al. [63] and Popović et al. [64]. Lyophilized samples were used for HPLC analysis; they were dissolved in 100% methanol, shaken at 120 rpm for 4 h, after which the supernatant was filtered through 22 μm nylon syringe filters and used undiluted for analysis. Phenolic compounds were tentatively identified based on their chromatographic behavior and mass spectrometric (MS and MS^2^) data, through comparison with available standards and relevant literature sources. A total of 20 compounds were identified and quantified by LC-MS in all the extracts and examined in heatmaps.

### 2.8. FTIR Analysis

ATR-FTIR spectroscopy (SpectrumTwo, Perkin Elmer, Waltham, MA, USA) was used to analyze the samples: control samples of expanded and extruded polystyrene, frass of experimental insects, and samples treated with fungi, which contained fungal mycelia and polystyrene particles and fungal mycelia. The sample preparation procedure is described in detail in Section 2.3. Spectra were recorded in the range of 4000 to 400 cm^−1^, with a resolution of 8 cm^−1^. For each sample, the full FTIR spectrum was recorded with 16 accumulations, and the analysis was performed in three replicates.

### 2.9. Statistical Analyses

Two-factor ANOVA with a balanced design (n = 3) was used to examine the differences between the mean values of enzyme activities between individual treatments for the two fungal species and between the two fungal species within individual treatments.

To assess and visualize the similarity between FTIR spectral profiles of fungi cultivated on EPS and XPS, principal component analysis (PCA) was employed as a multivariate technique for dimensionality reduction and pattern recognition. FTIR spectra were initially recorded in the transmittance mode and subsequently transformed into absorbance to enhance interpretability and ensure linearity concerning the concentration, according to the Beer–Lambert law. For the analysis, two specific spectral regions were selected: 400–1800 cm^−1^ and 2700–3700 cm^−1^. The intermediate region (1800–2700 cm^−1^) was excluded due to a lack of significant spectral variability, rendering it uninformative for multivariate analysis. Before PCA, spectral pre-processing was applied to improve data quality and comparability. Baseline correction was performed using the rubber band method to minimize baseline drift.

PCA was conducted on the pre-processed spectra, where principal components were computed as linear combinations of the original spectral variables. The eigenvectors (loadings) were derived to maximize the captured variance in the dataset, with each subsequent component orthogonal to the preceding ones. The first two principal components (PC1 and PC2), explaining the largest portion of total variance, were used to visualize the distribution and grouping of samples in a two-dimensional space. All PCA computations were performed using the *Orange Data Mining Toolbox for Data Analysis*, version 3.35 [65]. Graphical representations of PCA results were generated using Microsoft Excel (Microsoft Corporation, One Microsoft Way, Redmond, WA, USA, Version 365).

## 3. Results

The majority of *Tenebrio molitor* larvae successfully survived until the end of the experiment (some transforming into the imago stadium), feeding on two types of polystyrene foam as the only food source. Larvae were allowed to feed on polystyrene for 45 days, which was enough to collect sufficient amounts of frass with incompletely decomposed polystyrene for the analysis and setting up experiments with white rot fungi.

Fungal strains’ taxonomical determination was confirmed by the analysis of the ITS regions of their DNA. The amplified ITS regions sequences revealed 99–100% identity with different sequences of *Trametes versicolor* and *Pleurotus eryngii*, respectively, and were deposited in the NCBI database (accession numbers PV682683 and PV682684).

### 3.1. Ligninolytic Enzymes Activity Analysis

**Figure 3:**
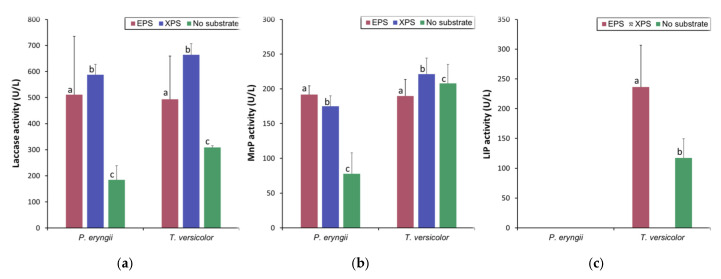
The enzyme activities measured in the media of *Pleurotus eryngii* and *Trametes versicolor* ((**a**) laccase activity in fungal mycelia; (**b**) Mn-peroxidase activity in fungal mycelia; (**c**) lignin peroxidase activity in fungal mycelia; EPS—expanded polystyrene, styrofoam; XPS—extruded polystyrene, styrodure). a, b, c are statistical classes indicating statistical significance of the differences between enzyme activities.

Mycelial growth was successful and abundant across all experimental treatments. The activity of the examined ligninolytic enzymes was not detected in controls containing polystyrene, making it clear that the detected enzyme activity originates from fungi. The measured values of enzyme activity are shown in Figure 3. High levels and similar patterns of laccase activity were recorded in the media of both strains of white rot fungi used in the experiment. The significantly lower Lac activity was registered in the treatment without a substrate compared to EPS and XPS, but there was no significant difference between the polystyrene treatments for both species (Figure 3a). Both species had high MnP activity values, but activities of the MnP of *T. versicolor* were significantly higher in XPS treatment than with *P. eryngii* (Figure 3b) and, especially, in the control samples compared to *P. eryngii*. Activities of both enzymes in the control with *T. versicolor* were significantly higher than in the control with *P. eringii*. LiP activity was detected only in the species *T. versicolor* in the absence of substrate and the treatment with EPS, but there were no significant differences between these two treatments.

### 3.2. UHPLC/Orbitrap MS Analyses of the Potential Products of Plastic Degradation

**Figure 4:**
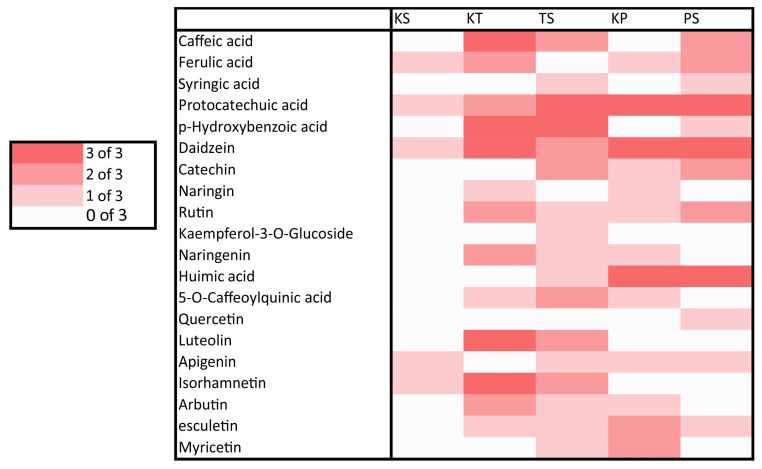
Detection frequency of phenolic compounds in methanolic extracts of *T. versicolor* and *P. eryngii* cultivated on larval frass derived from XPS. Detection was based on LC-MS analysis across three biological replicates per condition. Darker shades indicate consistent compound presence (3/3), while lighter shades and white reflect variable or absent detection.

**Figure 5:**
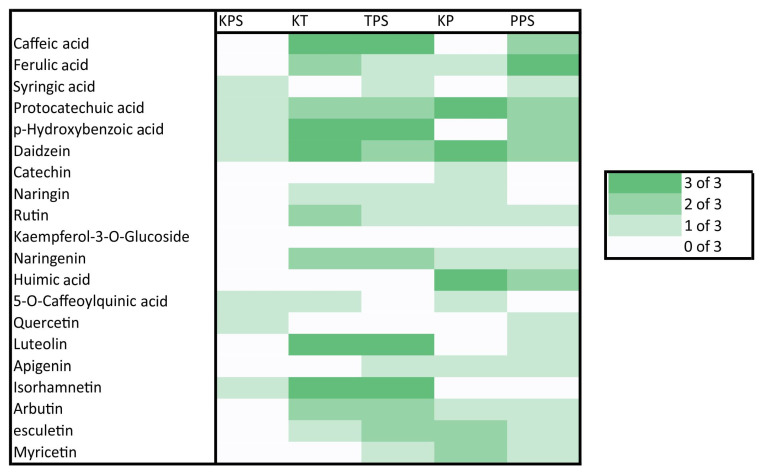
Detection frequency of phenolic compounds in methanolic extracts of *T. versicolor* and *P. eryngii* cultivated on larval frass derived from EPS. Detection was based on LC-MS analysis across three biological replicates per condition. Darker shades indicate consistent compound presence (3/3), while lighter shades and white reflect variable or absent detection.

Detection frequencies of phenolic compounds in the control (T.v.C for *T. versicolor*, P.e.C for *P. eryngii*) and treatments with XPS, styrodure (T.v.XPS/P.e.XPS), or EPS, styrofoam (T.v.EPS/P.e.EPS), are summarized in heat maps (Figure 4 and Figure 5).

Phenolic substances were detected in all treatments, but their profiles in the media with different polystyrene forms added expressed certain differences (Figure 4 and Figure 5). In the control mediums with added EPS or XPS only, small amounts of phenolic substances were detected sporadically. Ferulic and protocatechuic acids, daidzein, apigenin, and isorhamnetin were present in the media with ESP, while in media with XPS, apigenin was absent, and additionally, hydroxybenzoic, 5-O-caffeoylquinic acids, and quercetin were recorded. Phenolic profiles of the media of the two fungal species differ significantly (Figure 4 and Figure 5). Caffeic and p- p-hydroxybenzoic acids, naringenin, luteolin, and isorhamnetin were strongly produced in every treatment with *T. versicolor*, while protocatechuic and humic acids and daidzein were characteristically strongly expressed in all treatments with *P. eryngii*. Syringic acid and quercetin appeared only in media with polystyrene added to fungal mycelia, while kaempferol-3-O-glucoside appeared only in media with EPS added to mycelia of *P. eryngii*. Other phenolics were detected in the media in varying patterns (Figure 4 and Figure 5).

### 3.3. FTIR Analysis of the EPS/XPS Exposed to the T. molitor, P. eryngii, and T. versicolor in the Experimental Treatments

The FTIR profiles of both types of PS used in the experiment, expanded and extruded, look almost identical, except for minor differences in the intensity of a few individual peaks. The observed absorption bands in the FTIR spectrum and band assignment of polystyrene controls are shown in Table 1.

**Figure 6:**
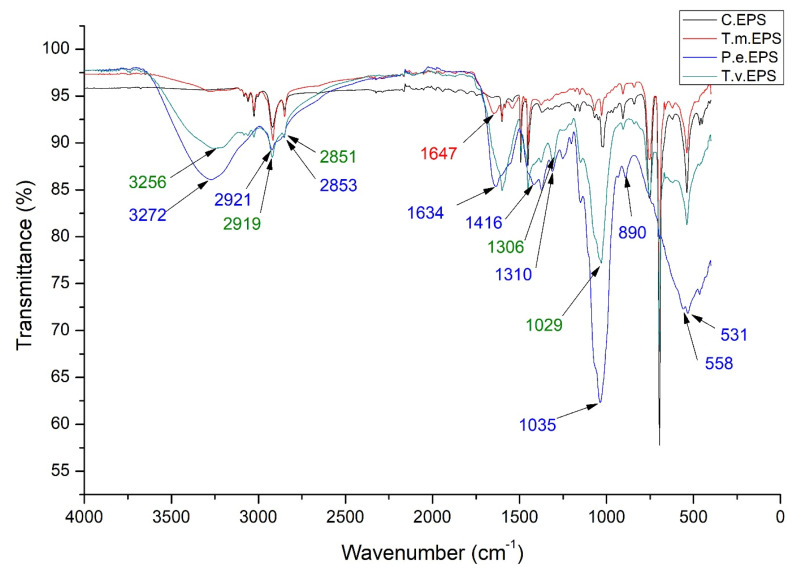
Average FTIR spectra of experimental treatments with expanded polystyrene (C.EPS—expanded polystyrene control; T.m.EPS—polystyrene after the digestive system of *T. molitor* larvae; P.e.EPS—polystyrene in insect frass treated with *P. eryngii*; T.v.EPS—polystyrene in insect frass treated with *T. versicolor*).

The FTIR spectrum of larval frass derived from expanded polystyrene and presented in Figure 6 (T.m. EPS) had a similar profile to the untreated polystyrene control. In addition to minor changes in the intensity of the peaks, one new peak that was not present in the control FTIR spectrum can be observed at 1647 cm^−1^ (Figure 6). In the FTIR spectra after incubation with two fungal species, significant differences can be observed, both concerning the control and polystyrene after passing through the digestive system of insects and to the two fungal species. After incubation with fungi *P. eryngii* (Figure 6, P.e.EPS), a loss of peaks at 3082, 3060, 3026, 1493, and 1452 cm^−1^, as well as the emergence of new peaks at 3272, 1634, 1416, 1310, and 558 cm^−1^ can be observed in the FTIR spectrum. Shifts in absorption peaks were evinced from 2917 to 2921 cm^−1^, 2848 to 2853 cm^−1^, 1028 to 1035 cm^−1^, 907 to 890 cm^−1^, and 538 to 531 cm^−1^. After incubation with *T. versicolor* (Figure 6, T.v.EPS), new peaks can be observed in the FTIR spectrum at 3256 and 1306 cm^−1^ and a shift in absorption peaks from 2917 to 2919 cm^−1^, 2848 to 2851 cm^−1^, and 1028 to 1029 cm^−1^.

**Figure 7:**
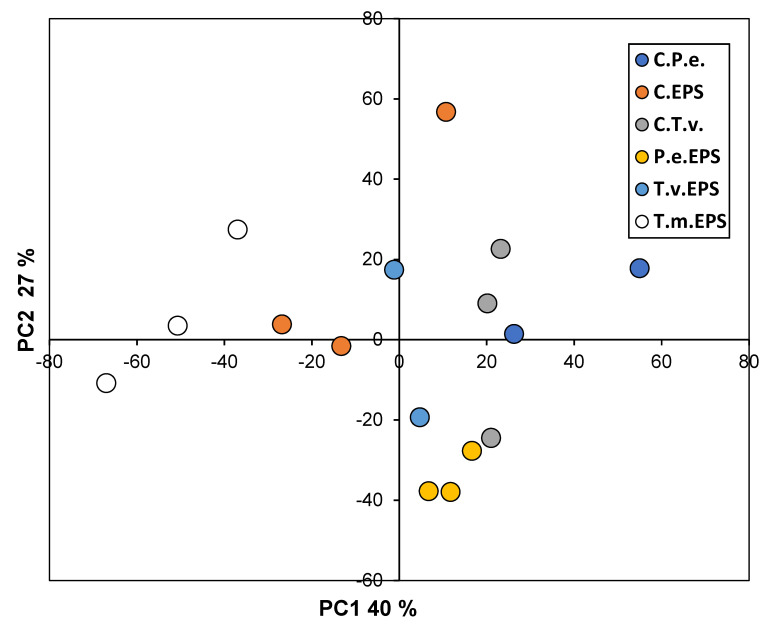
The score plot of the first two principal components (PC1 and PC2) obtained from PCA of FTIR spectra of treatments based on EPS The plot shows treatment distribution based on spectral similarity in the regions 400–1800 cm^−1^ and 2800–3700 cm^−1^. PC1 and PC2 together explain 83% of the total variance. (C.EPS—expanded polystyrene control; T.m.EPS—polystyrene after the digestive system of *T. molitor* larvae; P.e.EPS—polystyrene in insect frass treated with *P. eryngii*; T.v.EPS—polystyrene in insect frass treated with *T. versicolor*; C.P.e.—control of mycelia of *Pleurotus eryngii*; C.T.v.—control of mycelia of *Trametes versicolor*).

The PCA-based visualization of the relationships between the different spectra recorded by FTIR spectra of EPS biodegradation is presented in Figure 7. In this analysis, we introduced spectra of the fungal mycelia (Figure S1) to distinguish signals of fungi from those of plastic polymers (Figure S2). The clear differentiation of the spectra according to the treatment is visible. Spectra of *T. molitor*-treated EPS were the most similar to the EPS control, while spectra of fungal mycelia strongly differed from those of the EPS containing materials (differentiated with PC1, Figure 7).

**Figure 8:**
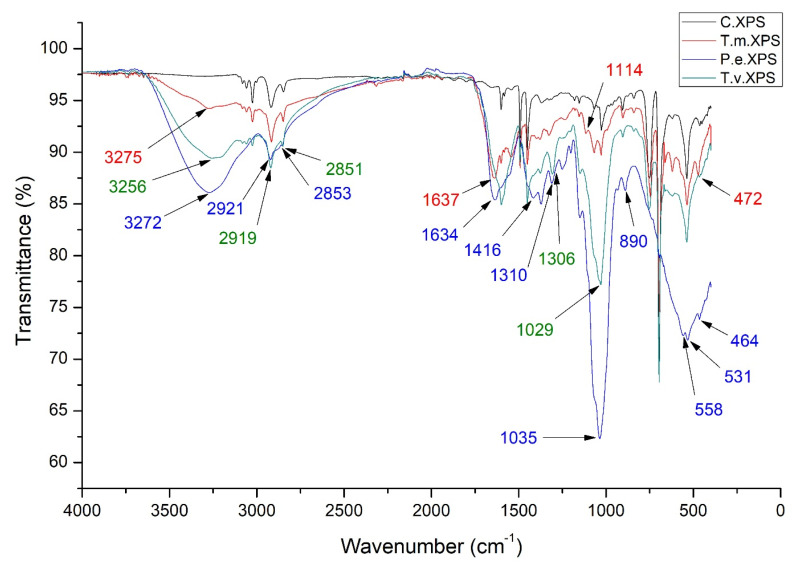
Average FTIR spectra of experimental treatments with extruded polystyrene (C.XPS—untreated extruded polystyrene control; T.m.XPS—polystyrene after the digestive system of *T. molitor* larvae; P.e.XPS—polystyrene in insect frass treated with *P. eryngii*; T.v.XPS—polystyrene in insect frass treated with *T. versicolor*).

The FTIR spectrum of larval frass derived from extruded polystyrene is presented in Figure 8.

Compared to the control, the largest differences in the obtained FTIR spectra of EPS after passing through the digestive system of insect larvae are the appearance of new peaks at 3275, 1637, 1114, and 472 cm^−1^ (Figure 8, T.m.XPS). In the incubation treatment with the species *P. eryngii* (Figure 7, P.e.XPS), the loss of peaks was observed at 3026, 1493, and 1452 cm^−1^, the appearance of new peaks at 3272, 1634, 1416, 1310, 558, and 464 cm^−1^, as well as the shift of peaks from 2917 to 2921 cm^−1^, 2848 to 2853 cm^−1^, 1028 to 1035 cm^−1^, 907 to 890 cm^−1^ and 538 to 531 cm^−1^, similar to the treatment with expanded polystyrene. In the experimental treatment with the species *T. versicolor* (Figure 8, P.e.XPS), the following changes were observed in the FTIR spectrum of extruded polystyrene: the emergence of new peaks at 3256 and 1306 cm^−1^ and shift in absorption peaks from 2917 to 2919 cm^−1^, 2848 to 2851 cm^−1^, and 1028 to 1029 cm^−1^, as also detected in the treatment with expanded polystyrene (Figure 8).

**Figure 9:**
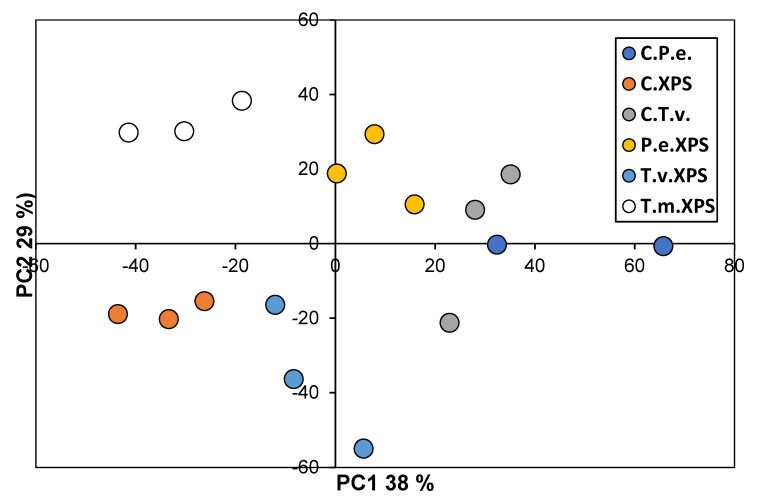
The score plot of the first two principal components (PC1 and PC2) obtained from PCA of FTIR spectra of treatments based on XPS The plot shows treatment distribution based on spectral similarity in the regions 400–1800 cm^−1^ and 2800–3700 cm^−1^. PC1 and PC2 together explain 83% of the total variance. (C.EPS—expanded polystyrene control; T.m.EPS—polystyrene after the digestive system of *T. molitor* larvae; P.e.EPS—polystyrene in insect frass treated with *P. eryngii*; T.v.EPS—polystyrene in insect frass treated with *T. versicolor*; C.P.e.—control of mycelia of *Pleurotus eryngii*; C.T.v.—control of mycelia of *Trametes versicolor*).

The PCA-based visualization of the relationships between the different spectra recorded by FTIR spectra of XPS biodegradation is presented in Figure 9. The spectra analyses showed even better differentiation of treatments with different biological agents along PC1, with PS treatments on one and fungal mycelia on the other side of the graph (Figure 9). It is also very visible that XPS degradation of T. molitor produced quite different outcomes than fungal treatments.

## 4. Discussion

In this contribution, we demonstrated that *Tenebrio molitor* larvae could feed on polystyrene materials, destroying their structure and transforming them into small particles (MPs) within their digestive tract. These particles still consisted of polystyrene molecules, but the signs of the polymer’s destabilization were evident. When offered to the two white rot fungal strains as a substrate, these particles were further degraded by the activities of laccases and Mn peroxidases produced by both fungi, resulting in the excretion of the various phenolic substances in the growth media.

*T. molitor* belongs to the order Coleoptera, family *Tenebrionidae*, and is a cosmopolitan species. Previous research indicated that polystyrene is susceptible to degradation in the intestinal tract of *T. molitor* larvae [70]. When feeding on polystyrene, the larvae nibble and chew PS, thereby mechanically breaking it down, which can be accepted as biodeterioration—the first step in its biodegradation. Further biodegradation takes place in the digestive tract of larvae through gut-microbe-dependent oxidative digestive machinery [70]. Among the gut microbes, *Enterococcus* and *Enterobacteriaceae* play a crucial role in this polystyrene biodegradation process [71]. In previously published papers, it was claimed that the development of *T. molitor* larvae to adulthood is impossible due to a lack of various nutrients, primarily nitrogen, but in our experiments, majority of larvae successfully survived feeding on polystyrene as its sole food source until the end of the experiment (45 days) and even completed their development into adult beetles. The research conducted by Peng et al. [70] indicates that the successful survival of *T. molitor* larvae feeding only on polystyrene is possible for about 4–5 weeks, which is similar to what we observed here.

It is well known that white rot fungi, like those used in experiments in this contribution, produce ligninolytic enzymes that are characterized by low substrate specificity [25,26]. High Lac activity was detected in the experiment with both strains of fungi and with both types of larval frass (Figure 3a). Due to a relatively low redox potential [72], laccases can directly oxidize only some phenolic compounds, but the spectrum of their activity is significantly expanded through redox mediators. These are phenolic compounds of low molecular weight, such as hydroxycinnamic acids [73]. Some of the compounds of this group, caffeic and ferulic acids, have been detected via UHPLC/Orbitrap MS analysis (Figure 4 and Figure 5). Since the enzymes as well as phenolic substances were present in the control treatment with fungi without the addition of PS, it can be assumed that both fungi may produce Lac in the presence of growth factors in the media and accelerate the production of such mediators. Additionally, both fungi also exhibited high Mn peroxidase activity with both types of larval frass (Figure 3b). MnP can directly oxidize phenolic compounds [74] but cannot directly oxidize non-phenolic aromatic structures of lignin [75]. Mn^3+^ cations, which are formed as a product of the catalytic activity of Mn peroxidases, are stabilized by forming chelates with various organic acids, such as oxalic acid, which are derivatives of fungal metabolism. These chelates of low molecular weight function as highly reactive redox mediators that non-specifically attack and oxidize different polymers [76]. Both Lac and MnP cause similar reactions when exposed to artificial polymers—they attack them by causing redox reactions, which produce ROS, and then oxidize functional groups within the polymer, thereby altering their structure to a more destructible state [77]. Further exposure of the polymer to these enzymes and generated ROS will cause its fragmentation, generating smaller structures with polar functional groups that are more reactive with enzymes [34]. Small products of such degradation can be transported into the cells for further metabolism [78].

LiP is an enzyme that has a high redox potential and can oxidize non-phenolic and phenolic aromatic compounds [79]. A typical substrate of lignin peroxidase is veratryl alcohol, which is produced as a derivative of the secondary metabolism of fungi [80]. This compound, through the mechanism of free radical production, can serve as a mediator of the oxidation of various compounds that are otherwise not substrates of lignin peroxidase. Fungi from the genus *Pleurotus* sp. do not produce typical lignin peroxidase, which was confirmed in our experiments (Figure 3c); instead, they produce versatile peroxidases [81]. LiP was detected in the *T. versicolor* medium in the absence of substrate and the treatment with EPS but, surprisingly, not in the treatment with larval frass derived from XPS. It could be possible that the firm structure of XPS or some additives blocked the production of LiP by *T. versicolor* (Figure 3c)

Obtained UHPLC/Orbitrap MS analysis data reveal that XSP frass strongly stimulates the biosynthesis of key hydroxycinnamic acids—caffeic and ferulic—in *P. eryngii*, whereas controls show no such induction. The selective emergence of syringic acid under ESP and XSP treatments (Figure 4 and Figure 5) suggests PS induced radical-scavenging pathways. Protocatechuic and hydroxybenzoic acids’ consistent detection across all *T. versicolor* conditions suggests a basal role in reactive-oxygen-species detoxification [82]. Flavonoid profiling indicates that quercetin production was detected in the control as well as in the media of *P. eryngii* with (XPS), confirming its role as a redox modulator included in PS degradation. Naringin moderate stimulation with EPS across treatments reflects activation of specific biosynthetic branches. The presence of EPS induced kaempferol-3-O-glucoside in *T. versicolor* treatment points to interspecies variation in flavonol glycosylation [83]. Finally, the detection of daidzein, apigenin, and esculetin in all fungal treatments suggests activation of fungi-specific metabolic pathways. Specific induction of chlorogenic acid across treatments emphasizes its species-specific antioxidant function, whereas the detection of arbutin and humic acid in species species-specific manner confirms their probable negligible role in plastic degradation. Overall, *T. versicolor* demonstrates a more robust induction of core antioxidant phenolics in response to XSP frass, suggesting these compounds as biomarkers of successful PS and XSP degradation and highlighting opportunities for optimizing fungal biodegradation through strain selection or engineering. The phenolic profiles correspond to the enzymatic profiles, confirming species-specific pathways for degradation of aromatic polymers.

The obtained FTIR spectra of larval frass derived from EPS were similar to the spectra of polystyrene in the control, while minor differences in peak intensity and the appearance of one new peak at 1647 cm^−1^ were detected. The FTIR spectrum of larval frass derived from extruded polystyrene is similar, with several new peaks at 3275, 1637, 1114, and 472 cm^−1^. Peng et al. [70] indicated that the peak at 1650 cm^−1^ represents −C=C− stretch and detected peaks at 1637 and 1647 cm^−1^, probably representing the same functional group. The peak at 3275 cm^−1^ is part of the 2500–3500 cm^−1^ area that is associated with the hydrogen bond of hydroxyl groups and/or carboxylic acid groups and suggests a shift from hydrophobic to more hydrophilic surface properties [84]. The incorporation of oxygen functional groups is considered one of the first steps and key evidence of biodegradation of polystyrene polymers [84]. According to Olmos et al. [66], bands in the region from 3200 to 2800 cm^−1^ can be assigned to C–H stretching vibrations. Bands at 3082, 3060, and 3026 cm^−1^ correspond to absorptions from the C–H stretching vibrations in benzene rings, while the bands at 2917 and 2948 cm^−1^ come from asymmetric and symmetric stretching vibrations from CH_2_ groups in the main polystyrene chain. The absorptions at 1601 and 1493 cm^−1^ come from C–C stretching vibrations in the aromatic rings. According to Bhutto et al. [67], band at 1452 cm^−1^ can be assigned to C–H deformation vibration of CH_2_ groups. According to Ilijin et al. [69], the bands in the regions from 1066 to 1028 cm^−1^ and from 755 to 696 cm^−1^ can be assigned to C–H bending vibrations of the ring in plane and C–H out-of-plane bending vibrations of the benzene ring. The main absorption peaks in these regions are observed at 1028, 907, 755, and 696 cm^−1^.

Principal component analysis (PCA) score plots based on FTIR absorbance spectra of fungal samples grown on polystirene were able to distinguish clearly between the polymer structures of different treatments (Figure 7 and Figure 9). As expected, samples from the C.P.e. and C.T.v.—the spectra of fungal mycelia exhibited the highest scores along PC1, indicating pronounced spectral differences from the other treatments, which are more closely clustered along this axis. Interestingly, spectra of *T. molitor* treatment exhibited the lowest scores along PC1, including C.EPS, indicating a very different mechanism of PS destruction in comparison to fungal treatments (Figure 7). Fungal treatments, on the other hand, differed significantly from the C.EPS, as well as from fungal controls (Figure 7). The pattern in the distribution of spectral profiles for the control and treatments with extruded polystyrene (XPS) showed similarities to that observed in the case of EPS treatments. However, PC2 made a clear differentiation between T.m.XPS and C.XPS, as well as between P.e.XSP and T.v.XSP. The observed lack of LP activity in *T. versicolor* treatment with XSP (Figure 3c) may be an explanation of such differences and the proximity of T.v.XSP spectra to C.XSP, as well as a difference between the spectra of the two fungal treatments.

In general, PCA of the FTIR spectra of different treatments revealed very clear differentiation between spectra produced by the PS molecule after *T. molitor* deterioration of original PS products into PS microplastics, fungal mycelia, and treated EPS and XPS (Figure 7 and Figure 9). Comparing the spectra of PS material before *T. molitor* treatment (Figure S1) and mycelia (Figure S2), it is easy to spot the differences between spectra that are visible in Figure 6 and Figure 8. These graphs are clear evidence that *T. molitor*, as well as both fungal strains, can degrade PS, but in species species-specific manner.

## 5. Conclusions

PS of different shapes can deteriorate and transform into the particle size of microplastics by *Tenebrio molitor* larvae in a relatively short time (4–6 weeks). Such particles can be degraded by white rot fungi through their enzymatic systems used for lignin degradation, like Lac, MnP, and LiP. These enzymes are known to form films on the polymer surface, eroding it through the oxidation of external functional groups and creating further ROS that attack the polymers. These new formations are susceptible to further action by oxygenases in a chain reaction. Thus far, this process has not led to the successful degradation of entire polymers, but most laboratory experiments, including ours, likely do not last long enough to enable such outcomes. For example, experiments lasting 50 days revealed stronger PS degradation by other white rot fungi [85]. It can be hypothesized that adding certain molecule accelerators, like those produced in our fungal media (caffeic and ferulic acids, coniferyl alcohol, etc), may speed up the process. Our results indicate that *T. versicolor* and *P. eryngii* employ similar, but not identical, enzymatic mechanisms, suggesting that including more than one fungal species in the process may enhance its efficiency. Even though we present very early-stage results of the combination of different organisms in the biodegradation of PS, optimizing this process could lead to complete PS degradation.

## Figures and Tables

**Table 1:** Observed absorption bands in the FTIR spectrum and band assignment of polystyrene controls.

Wave Number (cm^−1^)	Band Assignment	References
3082	Aromatic C–H stretching vibrations	Olmos et al. [66]
3060	Aromatic C–H stretching vibrations	Olmos et al. [66]
3026	Aromatic C–H stretching vibrations	Olmos et al. [66]
2917	C-H bond stretching	Meenashi et al. [67]
2848	Symmetric stretching vibrations of methylene groups –CH_2_	Olmos et al. [66]
1601	C-C stretching vibrations in the aromatic ring	Olmos et al. [66]
1493	C-C stretching vibrations in the aromatic ring	Olmos et al. [66]
1452	C–H deformation vibration of CH_2_	Bhuto et al. [68]
1028	The in-plane C–H bending of the phenyl ring	Olmos et al. [66]
907	Out-of-plane C–H bending of the phenyl ring	Olmos et al. [66]
755	Out-of-plane C–H bending of the phenyl ring	Ilijin et al. [69]
696	Out-of-plane C–H bending of the phenyl ring	Ilijin et al. [69]

## Data Availability

All data are available in the manuscript or Supplementary Data.

## Supplementary Materials

The following supporting information can be downloaded at: https://www.mdpi.com/article/10.3390/polym17131772/s1, Figure S1: FTIR spectra of fungal mycelia (CP.e—*P. eryngii*; CT.v—*T. versicolor*); Figure S2: FTIR spectra of expanded (CEPS) and extruded polystyrene (CXPS).

## Abbreviations

The following abbreviations are used in this manuscript:

PS	Polystyrene
ESP	Expanded Polystyrene
XPS	Extruded Polystyrene
Lac	Laccase
MnP	Manganese Peroxidase
LiP	Lignin Peroxidase
