# Supplementary material for: Tenebrio molitor Could Be an Efficient Pre-Treatment Bioagent for Polystyrene Initial Deterioration and Further Application of Pleurotus eryngii and Trametes versicolor in Microplastic Biodegradation
Source: Polymers (Basel). 2025 Jun 26;17(13):1772. doi: 10.3390/polym17131772 (PMC12251591; doi:10.3390/polym17131772)
Supplement: Supplementary file 1 [file polymers-17-01772-s001.zip › polymers-3702472-supplementary.pdf]

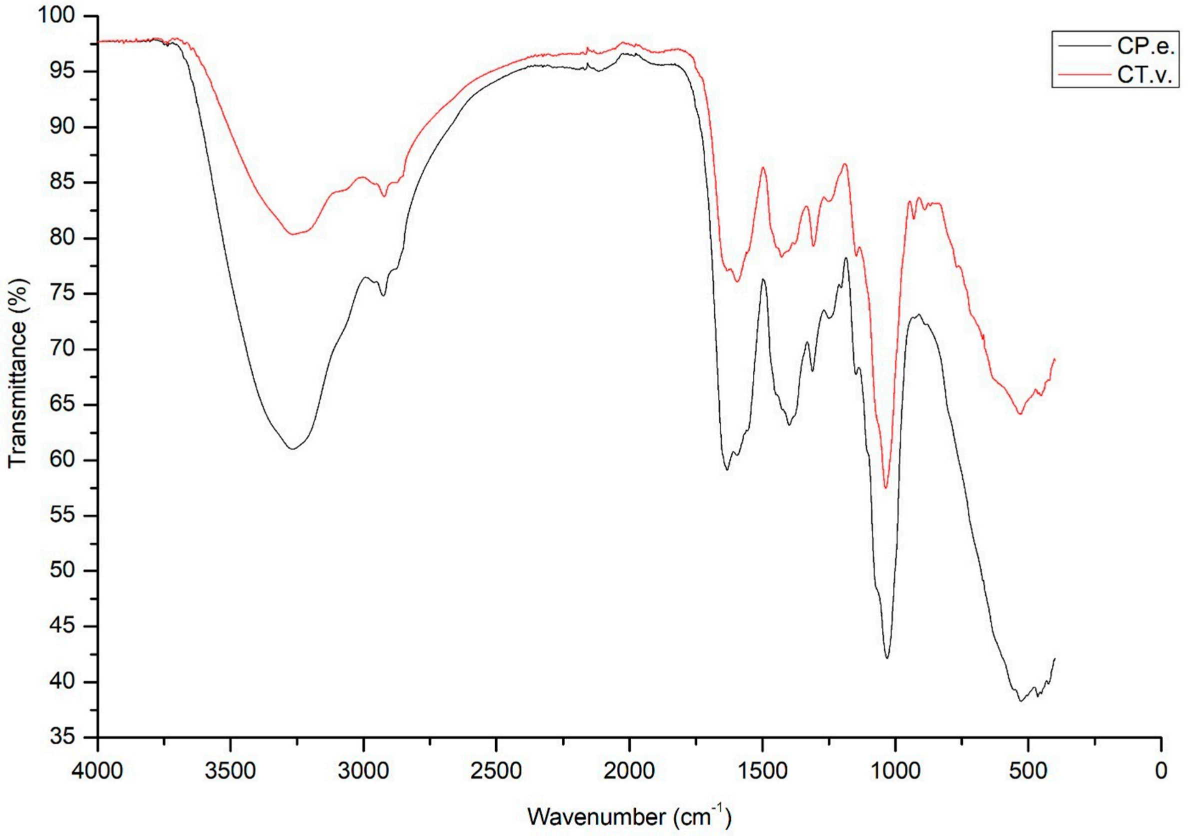

Figure S1. FTIR spectra of fungal mycelia (CP.e - *P. eryngii*; CT.v – *T. versicolor*).

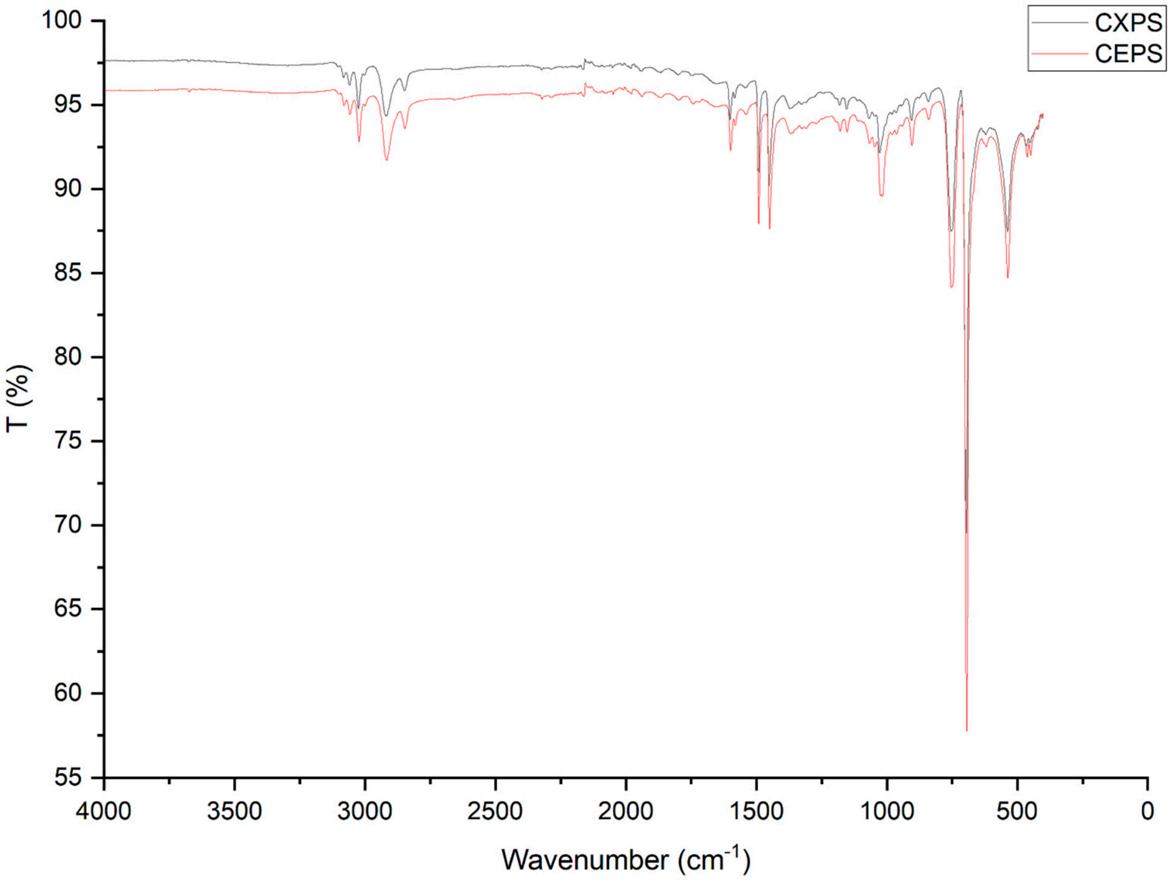

Figure 2S. FTIR spectra of expanded (CEPS) and extruded polystyrene (CXPS)
